# The unaffordable and the sublime

**DOI:** 10.1007/s11007-022-09567-y

**Published:** 2022-04-02

**Authors:** Shaun Gallagher

**Affiliations:** 1 Department of Philosophy, Philosophy, University of Memphis (USA), Clement Hall 337, Memphis, TN 38152 USA; 2 SOLA, University of Wollongong (AU), Wollongong, Australia

**Keywords:** Affordance, Awe, Aesthetic experience, Unaffordability, Sublime

## Abstract

In this paper I examine a set of exceptional aesthetic experiences that remove us from our pragmatic everyday life and involve a specific type of unaffordability. I then extend this notion of unaffordability to experiences of awe and its relation to the sublime. My analysis is guided by considerations of the phenomenologically inspired enactivist approach that supports an affordance-based accounts of aesthetic experience. I review some recent neurophenomenological studies of the experience of awe, and I then sketch out a phenomenology of awe as it approaches the sublime.

## Introduction

Contemporary approaches to embodied cognition, or what is sometimes called 4E (embodied, embedded, extended, enactive) cognition, which draw heavily from phenomenology, are often framed in pragmatic terms. On such approaches the lifeworld is conceived in relational terms of Husserl’s “I can,” or Heidegger’s *Zuhandenheit*, or Merleau-Ponty’s motor intentionality. Appeal is frequently made to Gibson’s notion of “affordance,” which was influenced by this phenomenological tradition, especially his reading of Merleau-Ponty.^1^ We are in-the-world in the mode of action-possibility, where our primary form of lived space is the peripersonal area of the reachable space that surrounds us, extended in some cases by our use of tools.^2^ Moreover, our primary form of lived time coheres on the integration scale around actions like reaching and grasping, or intentional structures of perceiving or intending a near-term proximate goal. This is the immediacy of everyday life.

On the one hand, this pragmatic immediacy can be broken or challenged in numerous ways. Illness or disability can transform the field of affordances or skew our lifeworld toward a different set of possibilities.^3^ Grief or depression can wipe out the social solicitations that usually motivate us, or bother us, to the point of interaction. Anxiety can make the world seem alien and unfriendly. Attesting to the relational nature of the lifeworld, external forces (the effects of war, poverty or racism), or material changes to our environments (natural disasters, climate changes), or disrupted social arrangements (as during a pandemic) can intervene in ways that subtract from the landscape of possibilities. On the other hand, and more positively, our lifeworld can be enhanced by our encounters with friends, or through learning or improving our skills, or simply by fortune. As Aristotle tells it, health, wealth, honor and friendship may expand our opportunities for happiness.

In this mix of good and bad, ups and downs, successes and failures there are a select class of experiences and practices that may strike us as exceptional, at least in the sense that they remove us, or are removed from pragmatic everydayness. One might think here of bodily practices such as meditation or yoga, but also of skilled actions, as found in athletics or the performing arts, or even gaming. One can also think of those activities that carry us into alternative realities, such as getting immersed in a novel, or movie, theatrical play, or concert.^4^ Here we encounter a diversity of aesthetic experiences, and we can ask how these experiences stand *vis a vis* our everyday pragmatic lifeworld. Specifically, we can pursue a phenomenology of such aesthetic experiences, as many have already done.

In this paper I want to pursue a phenomenological aesthetics of a specific kind, namely, one that explores the experience of awe and its relation to the sublime. My approach will be guided by considerations of the phenomenologically inspired enactive approach. I’ll start, in the first section, by looking at some recent embodied accounts of aesthetic experience that will allow us to set up the discussion of the experience of awe. In the second section I’ll review some recent neurophenomenological studies of the experience of awe. I’ll conclude, in the third section, by sketching out a phenomenology of awe as it approaches the sublime.

## An embodied-enactive aesthetics

We can see that there are some important differences between our experiences of the everyday world, where we encounter artifacts, tools and people, and our experiences of art, where we encounter, for example, depictions of artifacts, tools or people. As René Magritte writes on his painting, *The Treachery of Images*, “Ceci n'est pas une pipe,” we already know as we look at the painting that we cannot grab the pipe and smoke it. What Husserl calls the “I can” is necessarily different in regard to an artwork. Presented with a portrait, I can’t interact with the “person” in the painting in the same way that I can interact with a real person. And affectively, would my emotional response to the image of a tiger be the same as it would be if I were confronted by a real tiger? My pragmatic, affective, and intersubjective relations are all different in my encounter with art. Another way to say this is that the affordances that characterize my aesthetic experience are different from my everyday affordances. A real pipe affords smoking; the image of a pipe does not. A landscape offers a set of affordances or non-affordances for physical movement, a landscape painting does not. At the very least we should say that the image or artwork offers a different set of affordances – not smoking, not hiking, not social interaction – but what?

This is a difference in the way that we enactively respond to images or artistic representations. Elsewhere, I’ve argued that not all aesthetic experiences are the same. The aesthetic experience of the observer of an artwork, for example, in a museum, is quite different from the aesthetic experience of the dancer or the musician who is in the performance process.^5^ With respect to the observer of an artwork, however, I’ve suggested that aesthetic experience involves a “short-circuiting” of affordances, primarily thinking of motor or action affordances.^6^ It may also involve a “re-routing” of affective affordances since it certainly seems possible that I can have an affective or emotional response to a particular artwork that is nonetheless different from any encounter I would have with some entity that is not a work of art.

If this enactive approach to characterizing our experience of art is not the full story, it does demonstrate how such experience is different from real-world, instrumental-motoric and affective encounters with others and with real objects. Aesthetic experience shifts something in the structure of the lifeworld in the sense that it closes down specific possibilities (short-circuits affordances). At the same time, it also opens up other possibilities and helps us to see something different in what is depicted. Richard Wollheim, in his analysis of painting, introduces the notion of a twofold experience in which the observer is attuned to a double aspect of depiction in art.^7^ He distinguishes an awareness of the content or *what* is represented, and the technical way, or the *how* something is represented. Rather than thinking of the Wollheimian twofold as reflecting an externalist position (the twofold as the content portrayed plus the technical quality of the artwork),we can reinterpret this formulation phenomenologically, motivated by an analysis of the aesthetic experience of the performer, as a double attunement: an experience of the artwork, which is at the same time a self-awareness of experiencing the artwork.^8^ In this twofold consciousness, as a performer, I have a differential awareness of *what* is being performed (the music, the dance, the play, etc.) and *how* (or *how well*) I am performing it; or as an observer, an awareness of the artwork and of what it affords relative to my possible experience.^9^ I know, for example, not only that the artwork portrays a pipe, or a person, or a tiger, but also that I am not face-to-face with what is represented. Although I “see-in” the painting the character (or subject-matter) portrayed, this involves a modulation of my affordances. I think this is consistent with Wollheim’s view since he notes that these are “two aspects of a single experience that I have... two aspects [that are] distinguishable but also inseparable. They are two aspects of a single experience, they are not two experiences.”^10^ Built into this twofold attunement, in the case of observing art, there is the realization that my motoric and affective affordances are cut short, blocked or re-routed. I’m brought up short of being able to interact with the represented content, at the same time that I may have a sense of how I might engage if the figures were real rather than representations. Possibilities are opened up as possibilities, and at the same time, as impossiblities.

In this respect, as an embodied perceiver, I experience the work of art in the mode of an anticipatory kinaesthetics that I can never fulfill or satisfy in the way I would be able to satisfy my expectation if the object or the person were present and not just represented.^11^ One might say that the work of art falls short of actuality; or, perhaps more positively, the work of art transcends actuality. It presents me with enactive possibilities that cannot be readily actualized in the aesthetic encounter, without going through some further process – that is, without moving outside of the representational frame, e.g., by finding the actual person portrayed in the painting and interacting with her, or by engaging in some activity inspired by the work of art. I think this is likely even more the case with non-representational art.^12^

To be clear, it is not that the work of art does not offer me some set of affordances, it’s just that the typical affordances I might have for action, or a specific set of affective experiences, are short-circuited. What is presented in the artwork may offer new affordances for my imagination, relative to my observational skill. In addition, of course, an artwork presents me with other possibilities that could be actualized with the physical art piece itself. For example, I could remove it from its current location and place it somewhere else; I could purchase or sell it, etc. These latter affordances, however, do not constitute an aesthetic aspect of experience.

I want to make clear a contrast between this kind of enactive approach to aesthetic experience, and any approach that comes close to making it equivalent to a specialized instrumental attitude, as one finds in theorists who have employed the extended mind paradigm in their analysis of art and aesthetic experience. For example, Joel Krueger suggests that.

we perceive [music] as a resource we can *use* to do different things, much the same way we perceive tools and technologies as resources that help us accomplish different tasks. Music, I suggest, is experienced as having *instrumental* value. And what I suggest further is that musical affordances are what specify the different sorts of things we can do with music.^13^

Although Alva Noë’s work may be characterized as a version of enactivism that emphasizes sensory-motor contingency, he also comes close to an instrumental account of art and aesthetic experience focused on tool use.^14^ He associates art with the notion of “strange tools.” Noë establishes a variation of active externalism in which artistic tools and practices resist our perpetual tendency to offload cognition onto them, and this resistance reorganizes and reveals the world and our more basic practices to us. On the one hand, one might read Noë’s insistence on art’s impractical “subversion of function,” as something closer to a short-circuiting of affordances, and in opposition to an active externalism that would frame art in terms of tool-based practicality.^15^ On the other hand, as Mohan Matthen suggests, Noë indicates that the function of some tools is to resist being useful as tools (art is a tool-that-is-not-a-tool), and that this resistance itself fulfills the purpose of the tool.^16^ This ambiguity is left unresolved in Noë’s work.

Still, there is some sense of a shift in lifeworld structure in Noë’s work. He divides human behavior into two categories: organizational activities and re-organizational practices. Noë explains that organizational activities are basic activities that are performed due to basic biological motivations, and out of habit.^17^ Organizational activities are linked to tool use. “Roughly, a tool (such as a computer or a hammer) is the hub of organized activity.”^18^ He includes conversation as an instance of basic and habitual interaction through which we establish a certain relationship with our world and others. In contrast to these organizational activities, Noë frames some human practices, notably art and philosophy, as *reorganizational* practices, through which we can reassess or gain a new understanding of some previously unnoticed feature of our organizational activities. The precise nature of the features reflected in these reorganization practices, and what these features are features of, remains unclear. But it is clear that artistic depictions constitute, for Noë, a reorganizational practice that allows us to rethink and change this more basic organizational activity. Again, however, if art is a tool, even a strange tool, in the service of reorganizing the lifeworld, this can easily fall back into an instrumental view.

That art short-circuits everyday affordances and allows us to see something in a way that does not reduce to gaining instrumental insight; that it restructures the lifeworld, but is not something we employ for purposes of reorganizing the lifeworld, can be seen in the embodied approach to aesthetic experience taken by Merleau-Ponty in his essay on Cezanne:

We live in the midst of man-made objects, among tools, in houses, streets, cities, and most of the time we see them only through the human actions which put them to use. We become used to thinking that all of this exists necessarily and unshakably. Cezanne's painting suspends these habits of thought and reveals the base of inhuman nature upon which man has installed himself. This is why Cezanne's people are strange, as if viewed by a creature of another species.^19^

To the extent that art suspends our habits of thought, it differentiates itself from our everyday encounters with others or with worldly things. It reveals something different, as “strange,” in a way that shakes and challenges our everyday attitudes. With respect to listening to music at a concert, Merleau-Ponty puts it in terms of entering a different space.

Music is not in visible space, music erodes visible space, surrounds it, and causes it to shift, such that these overdressed listeners – who take on a judgmental air and exchange comments or smirks without noticing that the ground begins to tremble beneath them – are soon like a ship’s crew tossed about on the surface of a stormy sea.^20^

Heidegger’s analysis also suggests something similar. Heidegger understands art, not as something ready-to-hand (an instrument to be used – which involves our primary and everyday way of being-in-the-world), and not as something present-at-hand (an object for cognition – a derivative way of regarding the world, mistaken as primary by philosophers like Descartes). Rather, Heidegger regards art as something revelatory of being – and specifically, we could say, revelatory of being-in-the-world itself – that is, revealing of our own possibilities – as well as, perhaps, impossibilities.^21^

Finally, consider Maria Brincker’s idea of the “aesthetic stance,” which is similar to the idea that aesthetic experience involves short-circuiting affordances.^22^ Building on the Kantian idea of a practical disengagement that accompanies image perception, she suggests that an image (painting or sculpture) not only has “different affordances, but affords a sort of a ‘halt’ to our own ongoing environmental interactions …. [P]erception of action as image content does not afford the perceiver an overt complimentary response beyond simply watching what is being presented.” We can say that this is still an engagement of the observer with the art, but as Brincker puts it, this is an engagement that is halted at “the edge of action.”^23^ This links to what she identifies as an asymmetry involved in the aesthetic stance.

[M]y hypothesis is that there are aesthetic affordances, which invite a disengagement of action response or [a] “non-goal-directed attitude.” Notably, most artistic media (images, sculptures, stages, writings, recordings etc.) seem to invite asymmetric, non-interactive modes of perception, in that the beholder perceives the beheld but not the other way around. The further suggestion is that this asymmetry and lack of reciprocity in the aesthetic affordances precisely invites a different kind of engagement.^24^

This is clearly the case, for example, when I encounter a painting of one or more people. There is asymmetry, since the painted people do not respond to anything I do. More positively, however, I can actively imagine many things having to do with them, even if they cannot respond to me. I can look into their eyes, even if they don’t gaze back or follow my gaze in any real sense.

My embodied-enactive perception of a painting or sculpture involves a kinaesthetic-anticipatory response to a non-realizable (non-practical, non-interactionable) affordance. It seems appropriate to think that this non-realizability implicit (or explicit) in the encounter with an artwork is somehow registered/recognized in one’s motor system, generating a feeling different from an encounter with real tools or other persons – not a priming for action or interaction, but for an experience that allows me to see what is possible and what is impossible. This kind of affordance short circuits – it does so in a way that can come back to me and make me aware of my possibilities, and does so, at the very least, in a way that disrupts my ordinary engagements.

Let me summarize.

1. In some types of aesthetic experience (especially involving observing) there is a short-circuiting of everyday affordances and a “re-routing” of affective affordances, so that my engagement is halted at “the edge of action.”
2. This constitutes a shift in the structure of the lifeworld. We are in-the-world in a different way, or in some cases we have entered into a different, alternative world of possibilities that remain mere possibilities (or impossibilities that cannot be realized). We might think of these as possibilities we can imagine but never act upon.
3. There is a twofold unitary awareness in which (a) we are attuned to the short-circuiting – the fact that something is depicted in a specific way that closes off everyday possibilities, and (b) we are attuned to what we can see in that depiction, which opens up a different set of possibilities.
4. This double attunement (which involves both perception and imagination) may unfold kinaesthetically, in kinaesthetic-anticipatory responses to what we see.
5. Art, however, is not an instrument or tool that we simply use to reorganize the lifeworld.

## Experiences of awe

I begin by stipulating a definition of awe.

Awe: a direct and initial experience or feeling when faced with something

amazing, incomprehensible, or sublime.^25^

This is a definition taken from a neurophenomenological study of awe and wonder experiences, based on astronaut reports during space flight. In this study my colleagues and I created a simulated space flight and, in follow-up phenomenological interviews, asked individual participants (n = 104) to describe their experience during the simulation. We correlated the results with EEG, fNIR and ECG data on neural activity and heart rate, and with the results of a battery of questionnaires about cognitive and motivational measures, as well as cultural practices. We were able to replicate all of the varied experiences reported by astronauts while viewing the earth from close-earth orbit on the International Space Station or the NASA shuttles. This is often referred to as the “overview effect.”^26^ We were able to define awe more precisely through a set of consensus categories of experience reported both by astronauts and our participants. These included 34 categories overall, covering awe, wonder (which we defined as “a reflective experience motivated when one is unable to put things into a familiar conceptual framework – leading to open questions rather than conclusions”) curiosity and feelings of humility.^27^ The categories that we associated with the experience of awe include the following:

- *Captured by view/drawn to phenomenon*: expressing the idea that the subject does not want the experience to end, or that the experience is so positive they are drawn back to it
- Experiences of *elation*
- *Experience-hungry*: the idea that the person’s interest in having these sorts of experiences is so strong that they just want more of it. So they proactively put themselves in a position to have such experiences.
- Feeling of being *overwhelmed*
- *Scale effects*: feelings of the vastness of the universe or of one’s own smallness or insignificance when faced with that vastness (this is also a category associated with the experience of humility)
- Experiences of *surprise*
- Experiences of the *sublime*

Other closely related categories clearly registered aesthetic experience: as when the report included poetic expression or straight-forward statements about the beauty of what they were seeing, which in some cases were linked to the sense of being overwhelmed. Here is just one example from the astronaut Greg Chamitoff’s in-flight journal.

Incredible! I just noticed we were approaching London around midnight GMT. I decided to turn off all the lights and set myself up for some hopeful night [photographic] shots. What an amazing, spectacular, incredible, mind blowing view! So for a moment I just stared at the incredible display of life below me. From there we flew across the rest of Europe in a few minutes and I was just overwhelmed with the beauty of our civilization as it was, splattered across the dark landscape.^28^

That Chamitoff “sets himself up” to have these experiences suggests the category of being “experience hungry.” This is not clear, however, since he could mean he is setting up his photography equipment. It does seem clear that he is surprised by what he sees, and has both an experience of being aesthetically overwhelmed and of being captured by the view. Chamitoff also expressed scale effects.

From these windows, the Earth is so obviously floating in an endless void, and the feeling that washes over you is the sense of scale of the universe. The feeling I got was one of recognition that we are living on a such a tiny island in a vast ocean.^29^

Feelings get rerouted or overwhelmed. There is at once a double attunement, in this case, a consciousness of something too large to comprehend, and a recognition of our own place within the whole. Returning to the idea that our everyday lifeworld experience can be characterized in terms of pragmatic and social affordances, the experience of awe, in contrast, like aesthetic experience, seems to short-circuit such affordances. At least momentarily, as Chamitoff indicates, he is stopped in his tracks. The experience of awe is the experience of the unaffordable. In outer space we are in-the-world in a different way, and we are faced with some impossibilities: we cannot reach out and grasp what we see; and we cannot take it all in.

In this case, there is some question about whether this kind of awe experience issues in kinaesthetic-anticipatory responses to what we see. Although our study does not give us direct evidence for this kinaeesthetic response, it does provide us with data on a rich set of physiological (interoceptive and neuronal) changes that modulate the way we are attuned to what we see.^30^ These include changes in the complex patterns of brain resonance (including lower alpha and increased beta neural oscillations during awe experiences), changes in interbeat interval (IBI) for heartbeat, parasympathetic activation, Skin Conductance Response, Blood Volume Pulse, and surface electromyography.^31^ Other physiological markers of the awe experience have been explored – namely, instances of piloerection – i.e., “goosebumps” and shivers.^32^

Our study was, as far as we know, the first scientific study of awe connected with the overview effect. Since then, however, a number of studies have followed up, providing further evidence concerning the nature of the awe experience. In one study the notion of accommodation is emphasized. Awe involves a sense of vastness (scale effects), but also the need for accommodation, “the urge to adjust mental frames according to new incoming information.”^33^ Need for accommodation can be taken as a measure of surprise. That there is an “urge” or an attempt to accommodate what cannot be accommodated seems right. In our own study we found that those participants who had strong beliefs in a deity, and who engaged in religious practices (as measured by the Brief Multidimensional Measure of Religiousness/Spirituality had less experiences of awe.^34^ We interpreted this using Keltner and Haidt’s model in which awe experiences do not depend on positive religiosity, and may be connected with the inability to accommodate an experience into one’s conceptual schema.^35^ In other words, subjects who indicate strong religiosity may be better able to accommodate the effects of the visuals that inspire awe experiences in others, or may have different expectations that reduce surprise and modulate what they experience.

I also note that both Gallagher et al. and Quesnel and Riecke showed positive correlations between ratings of humility and awe.^36^ This is related to the experience of scale effects, where one may feel small or insignificant compared to the vastness of the universe.

## From awe to the sublime

The least reported category, both in the astronaut journals, and in our experimental simulation, was the experience of the sublime. Indeed, there was only one astronaut and one simulation participant who made any direct reference to a feeling of the sublime (in contrast to 29 reports of scale effects, for example).^37^ It’s not clear, however, that we had a clear definition in our study of what counted as the sublime. On the idea that the sublime is what we experience when confronted with something that overwhelms even the imagination, we thought of it as a combination of feeling overwhelmed and experiencing scale effects.^38^ The kinds of things that can elicit such a combination, as Kant suggests, although not uncontroversially, are things in nature rather than in artworks.^39^ He adds that the natural things that may lead to feelings of the sublime are such that they do not involve the idea of purpose, and he gives the examples of mountains and the sea.^40^ More specifically, mountains, the sea, the stars, or in the case of our astronauts, the earth viewed from space, are not themselves sublime, according to Kant; the experience of such things is sublime, since, it “is not contained in anything in nature, but only in our mind.”^41^ From an enactive perspective, however, we should consider the sublime to be a relational concept – an experience, not *only* in our minds, but in our relation to something that entirely transcends the capacity of our minds.

We can also find a linkage to Kant’s idea that the experience of the sublime involves respect, and moral feeling since, as we found in our study of awe, both the experiences of being overwhelmed and of scale effects motivate a shift in moral perspective, as well as an association with humility.^42^ Many of the astronauts and many of the participants in our study, after experiencing the earth from space, express a new concern for the earth’s ecological health, and for peoples who are caught up in wars and natural disasters. Such concerns can motivate action in the long term, and can restructure our everyday thinking about our own behavior, and that of others.^43^

Perhaps the most important thing to be said about the sublime is what we cannot say. The notion of the sublime includes the idea that it is something that cannot be represented. According to Nathan Stormer,

A distinctive mark of sublimity is that it escapes representation, even invalidates communication, and by some accounts eviscerates the capacity of discourse to function. In a more everyday sense, it leaves us “speechless.” Sublimity has been powerfully associated with the limits of the subject in relation to the world: her or his incapacity to say what exceeds finite human perception and comprehension.^44^

The idea that the sublime somehow goes beyond discursive form or defies representation has been defended by numerous philosophers.^45^ It is also said to be a rare experience because the exceptional quality of the natural or technological arrangement that precipitates it is rare, or because one requires an exceptional frame of mind.^46^

On the one hand, the rarity of the sublime may explain why it did not often register in the various studies of awe cited above. On the other hand, the idea that it escapes discourse or defies representation suggests that there may have been more of a sublime experience than could be documented. This may link to the unaffordability of awe and the sublime. Not only does the sublime not afford action, it does not afford discursive expression. If it is not easy to prove this negative, there is nonetheless some evidence to be found in the phenomenological interviews following the near-earth-orbit simulations. Specifically, there were significant pauses while subjects searched for words they could not find to express what they experienced. During the interviews subjects would hesitate, stare away, clearly searching for words. In the transcripts these moments were signified by ellipses. Here are some examples.

I think it’s the vastness of really… to me, then I start thinking of how huge our universe is. Like, just looking at this and this is just a little part of what I’m looking at and how much more there is. That’s the part that I admired… The beauty of the lights and all that, but, to me, somebody created all that. That blows me away.

… There was, let me think of the word that I’m looking for… maybe that… that… you know… you feel bigger in the sense that when I’m in the world, when I’m down in Florida… You know, I feel like small. I’m just this small person in this big huge world. When I’m looking down on it, it made me feel kind of like “wow, I’m not that small.” Does that make sense?

I guess I thought it was really… really cool that we live on such a big planet, and that’s why I was kinda looking for where we are in relation to everything else.… I guess you would say… I was a little bit in awe of what I was seeing cause not many people get that view.

Even in every day life I like looking up and looking at the stars… I usually think about how far away they are and how tiny they look, and I know that they’re actually these huge… beings… I was thinking about how many there were, and… I kind of thought about how far away we were since we’re on that space shuttle. How far we would be in comparison to like… If we were standing on the Earth. … I thought it was really nice. I’m trying to think of a better way to describe it.

It was just like… For me, it was like what I normally feel when I think of Earth from this perspective and also at that moment… Was… It is a vast amount of space that we are not going to be able to, as a species of homo sapiens, we’re not going to be able to identify and figure out everything… Even though we try to, there’s just too much going on and the Earth is always gonna have its secrets…Yeah, it was just like this… You feel so small.

Frequently the ellipses, which here signify varying amounts of time between words, fall around expressions of scale effects and how the subjects feel about such effects. Following our research methodology we attempted to capture the experience of awe in consensus categories derived from the astronauts’ journals. The positive data associated with the consensus categories and their related vocabulary, however, may not be the only kind of significant data available in the interviews. The stumbling and the hesitation of the subject at certain points during the interviews may also be significant, precisely for those experiences that are difficult or impossible to represent. One feature of such experience may be its ineffability, which may be captured by looking for the gaps between concepts in the subject’s reports, the places where the concepts seem to be missing. Such an ellipsis in the transcripts could count as what the Greeks called, using the same term, *élleipsis*, an “omission” or “falling short.” Indeed, what is left unsaid, in these contexts, may be telling us something. Namely, that the unaffordability of the experience of the sublime extends even to our ability to express it.

## Conclusion

I’ve been tracing a specific type of unaffordability that increases by degree as one moves from aesthetic experiences of artistic depiction (as one might have when observing a painting in a museum), to experiences of awe (in the particular case of viewing the earth from space), and this extended into the experience of the sublime. Aesthetic experience as it is connected to our perception of the artwork involves a short-circuiting of everyday affordances, and perhaps a halting at the “edge of action.”^47^ This does not rule out alternative affordances that may be presented by the artwork. In the case of the awe experience, we find a similar short-circuiting with perhaps even less opportunity to engage in any immediate action in regard to what we experience, in some cases as a response to phenomena of the natural world, and to the extent that we are captured by the view and stopped in our tracks. And this seems even more the case when awe involves the sublime, where the unaffordability extends not only to action but to expression and our ability to say anything about it.

## Footnotes

1 See Chemero (2009); Heft (2001).

2 Maravita and Iriki (2004).

3 Rietveld and Kiverstein (2014).

4 Schutz (1974).

5 Gallagher (2021).

6 Gallagher (2011).

7 Wollheim (2015).

8 For the debate between internalism and externalism in aesthetics, see e.g., Shelley (2017) and Dickie (1965).

9 In this respect, we can, as John Carvalho (2019) suggests, think of the art observer as engaged in a skilled performance.

10 Wollheim (2015, p. 46).

11 It would require further analysis to unpack this point, but it is consistent with (1) Husserl’s (1997) analysis of the intrinsic kinaesthetics involved in perception, (2) the contemporary neuroscience of canonical and mirror neurons that activate when I perceive action possibilities (with things or other people, respectively) (see Freedberg and Gallese 2007), and (3) the enactive view that perception and action are closely coupled (Gallagher 2011; 2017).

12 This readily pertains to the aesthetic experience of painting and sculpture and to most performing arts from the perspective of the observer/audience. But not all aesthetic experience is the same in this regard. Elsewhere I’ve argued that it is not the case with respect to the aesthetic experience of performing artists as they engage in performance (Gallagher 2021). Architecture seems to me an exception.

13 Kreuger (2014); emphasis in the original; also see Cochrane (2008); Kersten (2017); Kersten and Wilson (2016).

14 Noë (2015).

15 Ibid, (p. 98).

16 Matthen (2016).

17 Noë (2015, pp. 7–8).

18 Ibid, (p. 19).

19 Merleau-Ponty (1964, pp. 15–16).

20 Merleau-Ponty (2012, p. 234).

21 Heidegger (1993).

22 Brincker (2015).

23 Ibid., (pp. 122–123). This concept of being “at the edge of action” is consistent with the notion of short-circuited affordances (Gallagher 2011) and in fact Brincker cites my account as such. She supports this idea by citing evidence from brain imaging studies that suggest activation of the default mode network (DMN) while viewing artworks (e.g., Vessel, Starr and Rubin 2013). Activation of the DMN correlates with awake resting states where the subject is not practically engaged with any particular task.

24 Brincker (2015, p. 123).

25 Gallagher et al. (2015).

26 White (1987).

27 Gallagher et al. (2015, p. 6).

28 In-flight journal, 11/15/08; cited in Gallagher et al. (2015, p. 11_.

29 In-flight journal, 11/15/08; cited in Gallagher et al. (2015, 48).

30 In our study it was necessary to limit actual movement in the simulation condition in order to minimize the complexity of EEG data. In a study by Chirico et al. (2018), where EEG was not involved, the experimenters allowed movement in the form of navigational interactivity to improve the sense of presence. This type of instrumental activity, however, could complicate the experience if it involves kinaesthetic processes not directly related to awe. Stepanova, Quesnel and Riecke (2019) also restricted movement; they document a feeling of disembodiment, but this may be an artifact of the VR setup in that experiment and not directly connected to awe experience. They also reported a lack of self-motion illusion for some participants in the space scenario, but again it is not clear whether this is connected with the experience of awe.

31 Gallagher (2015 et al., 93ff); Shiota et al. (2011); Chirico et al. (2017).

32 Stepanova, Quesnel and Riecke (2019); also Quesnel and Riecke (2018).

33 Chirico et al. (2018, p. 2).

34 Idler et al. (2003).

35 Keltner and Haidt (2003).

36 Gallagher et al. (2015, 126); Quesnel and Riecke (2018).

37 Stepanova, Quesnel and Riecke (2019), who coded their interviews using the consensus categories from Gallagher et al. (2015), indicate that their subjects did not report anything that fit with the category of the sublime. Quesnel and Riecke (2018), however, counted five participants as reporting “sublime/incomprehensible” experiences. The same number experienced feeling scale effects, but only two reported feeling overwhelmed. They provide this example of a report categorized as sublime/incomprehensible: “I can’t believe what I am seeing. Is that real? It feels real,” and one categorized as scale effects: “I can’t believe this can exist. It feels much greater than me.”

38 Gallagher (2015, p. 28).

39 See Ginsborg (2019)

40 Kant (1987, §26, pp. 252–253; 256).

41 Ibid. (§28, p. 264).

42 Ibid. (§27, p. 257 and §29, pp. 255–256, respectively).

43 Also see Stepanova, Quesnel and Riecke (2019).

44 Stormer (2004, p. 212).

45 Burke (1889); Hegel (1975); Kant (1987); Jameson (1991); Longinus (1991).

46 Stormer (2004, p. 215).

47 Brincker (2015)
